# The trans-Saharan slave trade - clues from interpolation analyses and high-resolution characterization of mitochondrial DNA lineages

**DOI:** 10.1186/1471-2148-10-138

**Published:** 2010-05-10

**Authors:** Nourdin Harich, Marta D Costa, Verónica Fernandes, Mostafa Kandil, Joana B Pereira, Nuno M Silva, Luísa Pereira

**Affiliations:** 1 Laboratoire d'Anthropogénétique, Départment de Biologie, Faculté des Sciences, Université Chouaïb Doukkali, El Jadida, Morocco; 2 Instituto de Patologia e Imunologia Molecular da Universidade do Porto (IPATIMUP), Porto, Portugal; 3 Institute of Integrative and Comparative Biology, Faculty of Biological Sciences, University of Leeds, Leeds, UK; 4 Medical Faculty, University of Porto, Portugal

## Abstract

**Background:**

A proportion of 1/4 to 1/2 of North African female pool is made of typical sub-Saharan lineages, in higher frequencies as geographic proximity to sub-Saharan Africa increases. The Sahara was a strong geographical barrier against gene flow, at least since 5,000 years ago, when desertification affected a larger region, but the Arab trans-Saharan slave trade could have facilitate enormously this migration of lineages. Till now, the genetic consequences of these forced trans-Saharan movements of people have not been ascertained.

**Results:**

The distribution of the main L haplogroups in North Africa clearly reflects the known trans-Saharan slave routes: West is dominated by L1b, L2b, L2c, L2d, L3b and L3d; the Center by L3e and some L3f and L3w; the East by L0a, L3h, L3i, L3x and, in common with the Center, L3f and L3w; while, L2a is almost everywhere. Ages for the haplogroups observed in both sides of the Saharan desert testify the recent origin (holocenic) of these haplogroups in sub-Saharan Africa, claiming a recent introduction in North Africa, further strengthened by the no detection of local expansions.

**Conclusions:**

The interpolation analyses and complete sequencing of present mtDNA sub-Saharan lineages observed in North Africa support the genetic impact of recent trans-Saharan migrations, namely the slave trade initiated by the Arab conquest of North Africa in the seventh century. Sub-Saharan people did not leave traces in the North African maternal gene pool for the time of its settlement, some 40,000 years ago.

## Background

The recent high-resolution mtDNA studies are offering the possibility of shedding light on ancient and recent human migration events, allowing to inferring more precisely about the geographical origin of lineages observed nowadays in a certain region. In fact, the characterization of the full mtDNA sequence is being used to investigate local events as the Chadic expansion from East Africa towards Chad Basin in the last 8,000 years [1] or historic movements as the diaspora of Jews [2,3], which could not be approached in previous more limited mtDNA surveys.

This approach is being applied to the long-enduring discussion about pre-historic migrations across the Mediterranean Sea, leading to exchange of lineages between Iberia and Maghreb [4,5]. Recently, the sub-characterization of H-lineages observed in several North African populations revealed its affiliation within Iberian expanded lineages, after the Last-Glacial Maximum [6,7], being the same observed in Tuareg living in the Sahel [8]. The Near Eastern contribution to the pool of H lineages in North Africa was minimal, indicating that a pre-historic European lineage input occurred in elevated frequencies enriching the ancient Near Eastern background of North African populations mainly constituted by the low frequent haplogroups U6 and M1 [9].

Another major contribution to the pool of North African populations was the sub-Saharan one. It is known that a proportion of 1/4 to 1/2 of North African female pool is made of typical sub-Saharan lineages (designated as haplogroups L0-L6), in higher frequencies as geographic proximity to sub-Saharan Africa increases [4,5]. Nevertheless, the Sahara is a strong geographical barrier against gene flow, at least since 5,000 years ago, when desertification affected a larger region, ending up the humid and greening conditions established by around 10,000 years ago, in the so called Holocene Climatic Optimum [10].

**Figure 1:**
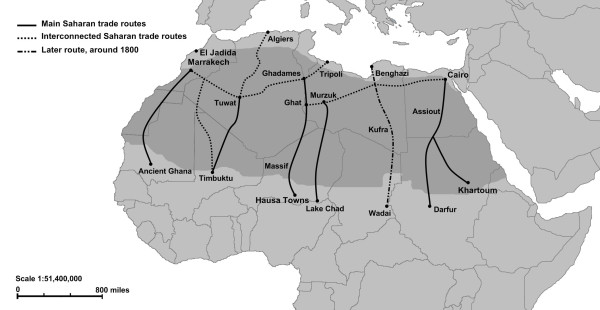
**Routes for trans-Saharan slave trade**. Adapted from Segal (2002) and Lovejoy (1983).

But, if geographical and climatic conditions have not been favorable to sub-Saharan gene flow to North Africa in the last 5,000 years, the Arab trans-Saharan slave trade could have facilitate enormously this migration of lineages. Till now, the genetic consequences of these forced trans-Saharan movements of people have not been ascertained, being over-shadowed by the Atlantic slave trade towards the New World. In fact, the huge number of sub-Saharan people introduced in the New World from the 16^th ^century onwards allowed to investigating in great detail the genetic consequences of this historical event [11-13], and the complete sequencing of L-lineages is indicating very precisely about the origin of lineages observed nowadays in America [14]. Nonetheless, some authors affirm [15] that the Arab slave trade of black slaves was much the same in total to the Atlantic slave trade, and interestingly far longer in the time scale. It began in the middle of the seventh century (650 A.D.) and survives still today in Mauritania and Sudan, summing up 14 centuries rather than four as for the Atlantic slave trade. Although estimates are very rough, figures are of 4,820,000 for the Saharan trade between 650 and 1600 A.D., and, for comparison purposes, of 2,400,000 for the Red Sea and the Indian Ocean trade between 800 and 1600 A.D. [16]. Notwithstanding the thousands of kilometers along the edge of the Sahara, the Red sea and the East African coast, from where slave exports came, there were relatively few export points, concentrating geographically the impact of the trade. Black slaves were brought by Berber and Arab merchants mainly to actual Morocco, Algeria, Libya and Egypt through six main routes that crossed the desert (Figure 1): one went north to Morocco from ancient Ghana (at present southeastern Mauritania and Western Mali); a second brought slaves to Tuwat (southern Algeria) from ancient Timbutku (Mali); a third passed from the Niger valley and the Hausa towns through the Air Massif to Ghat and Ghadames; a central route linked Lake Chad region to actual Libya (Murzuk), being one of the most important in slave commerce as it offered oases at regular intervals that could satisfy the caravan's needs; in East Africa, the slave caravan followed mainly the Nile River from actual Sudan (Dar Fur) to Egypt (Assiout); and a sixth passed north from the confluence of the Blue and the White Nile to Egypt. Some of these routes were interconnected: the routes north from Timbuktu went to Morocco, Algeria, and Libya; while the Dar Fur-Egypt route connected with the route north from the upper Nile valley.

Males were sought for a variety of functions: doorkeepers, secretaries, militaries or eunuchs. Black soldiers were seen from Islamic Spain to Egypt, and in Morocco a whole generation of black young boys were bought at the age of 10 or 11 and trained to become its army. However, the bulk of the trade was in females, as domestic servants, entertainers and/or concubines: two females for every male overall, in contrast to the ratio of two males for every female overall in the Atlantic trade [15]. Some harems could be enormous, reaching even the extravagating number of 14,000 concubines. Young female slaves were instructed in household crafts and were then provided with resources to buy a home and get married.

The Eastern sub-Saharan slave trade towards Arabia was investigated through mtDNA hypervariable region I (HVRI) diversity [17], leading to concluding that higher frequencies of L lineages are observed in Arab comparatively with non-Arab populations in the Near East, having been introduced in the last 2,500 years. These conclusions were supported afterwards by other studies [18,19]. This Eastern sub-Saharan slave trade involved mainly maritime routes across the Red Sea, which was dominated by the Southern Arabs, already around the 12^th ^century BC.

The Western trans-Saharan slave trade deserves a more careful genetic investigation. In this work we will present the results of mtDNA haplogroup affiliation of El Jadida population, approximately 100 km south of Casablanca, in the Moroccan Atlantic coast. We performed high-resolution screening of selected haplogroups in this Moroccan sample: haplogroup H, in order to get more evidence on North Mediterranean influence; and haplogroup L3, one of the most geographically diversified sub-Saharan haplogroup. For the L3 haplogroup, we conducted the complete mtDNA sequencing of 8 L3 haplotypes from El Jadida, and compared the complete North African L3 sequences which have been described [14,20-22] with the many other known sub-Saharan sequences (summed up in [23]). We also performed analyses of geographical interpolation for sub-Saharan haplogroup frequencies across Africa, by using an extended database summing up 4908 individuals.

## Methods

### Samples and DNA extraction

Blood samples were collected from 81 unrelated people from El Jadida, Morocco, nearly 100 km south of Casablanca. Appropriate informed consent was obtained from all individuals and total DNA was extracted from blood using a standard Chelex 100 protocol.

### mtDNA amplification and sequencing

The mtDNA hypervariable regions I and II (HVRI and HVRII) were amplified as described elsewhere [24], in both forward and reverse directions. The amplified samples were purified with Microspin S-300 HR columns (GE Healthcare, Uppsala, Sweden) and automated sequencing was carried out in an ABI Prism 3100 (AB Applied Biosystems, Foster City, CA, USA) using the kit Big-Dye Terminator Cycle Sequencing Ready Reaction (AB Applied Biosystems, Foster City, CA, USA). Temperatures profile for sequencing reactions consisted in denaturation at 96°C for 4 min and 35 cycles of 96°C for 15 s, 50°C for 9 s and 60°C for 2 min, followed by 60°C for 10 min. Sequence editing was performed both by using the BioEdit version 7.0.4.1 [25] and by manually checking the electropherograms, tasks performed by two independent investigators.

Haplogroup H variation was dissected in a total of 14 samples according to [26], which basically consisted in sequencing four mtDNA coding-region segments encompassing the principal diagnostic positions in haplogroup H samples: 3001-3360, 3661-4050, 4281-4820, and 6761-7050 (a total of 1580 base pairs). Furthermore, haplogroup L3 variation was investigated in 8 samples by performing complete sequence of the molecule (~16,569 bp) as described in [27], in a total of 32 overlapping segments of around 600 bp each. The 8 complete mtDNA sequences are deposited in GenBank database with accession numbers: GU455415-GU455422.

### Haplogroup affiliation

Mutations were scored relatively to the revised Cambridge Reference Sequence (rCRS; [28]), and its positions numbered from 1 to 16569. For haplogroup affiliation, the most recent phylogenetic data, including information from complete sequencing, were followed: for H [29]; for K [2]; for J, R, T, and V [30]; for U [30,31]; for I and M1 [32]; for X [33]; and for L [14].

### Statistical analyses

Analysis of population structure, molecular diversity measures, and tests of selective neutrality were executed in the software Arlequin version 3.0 [34].

Phylogenetic reconstruction of mtDNA sequences was based on HVRI and complete sequence. A preliminary network analysis [35] led to a suggested branching order for the tree and the L3 tree published in [14] was used as reference tree. The dates of the most recent common ancestor of specific subclusters in the phylogeny were estimated using ρ, the average number of transitions from the ancestral sequence type to all sequences in the cluster, based in the recently updated mutation rate published by [36] for the entire molecule (1 mutation in every 3624 years), and by using the calculator provided in the paper. The highly variable position 16519 was not considered for the time estimates. Each tip node of the phylogenetic tree was counted as one event if shared by a few samples.

**Figure 2:**
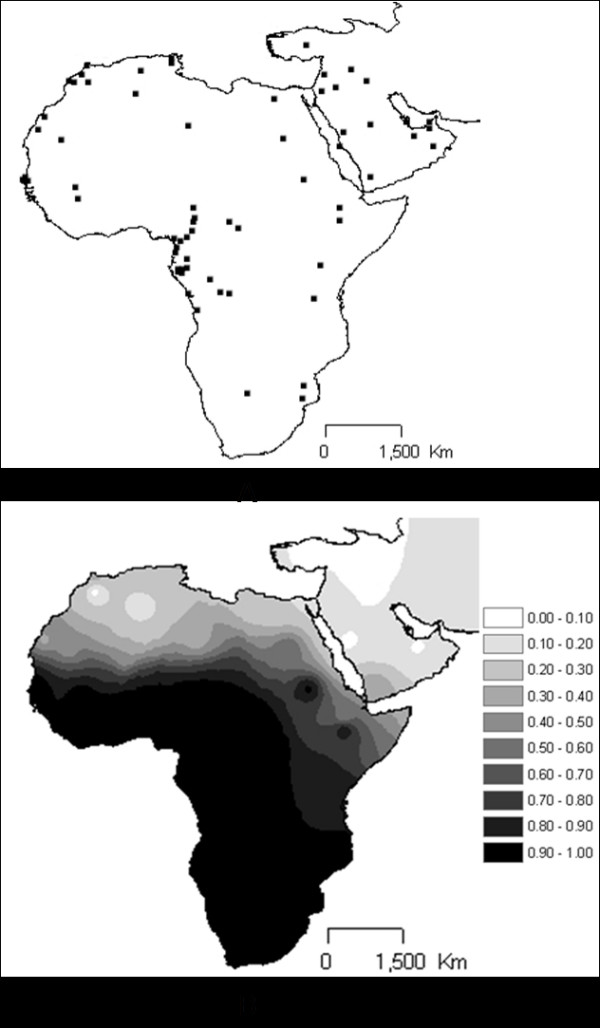
**Map showing location of the population samples (A) used in this work and interpolation map for the L lineages in those samples (B)**.

To determine and visualize the geographical distribution of haplogroups L interpolation maps were drawn by using the "Spatial Analyst Extension" of ArcView version 3.2 http://www.esri.com/software/arcview/. The "Inverse Distance Weighted" (IDW) option with a power of two was used for the interpolation of the surface. IDW assumes that each input point has a local influence that decreases with distance. The geographic location used is the centre of the distribution area, from where the individual samples of each population were collected. Data for other populations were taken from several publications and are summed up in Additional File 1 and displayed in Figure 2A.

Correlograms for Morans I indices versus distances were obtained for the total L in the populations and for L0, L1, L2 and L3 proportions of the sub-Saharan pool in the samples by using the PaSSAGE software v 1.0 [37]. The existence of a cline is assumed when a continuous decline trend composed of statistical significant points is observed.

## Results and Discussion

### mtDNA diversity and haplogroup affiliation in El Jadida sample

The characterization of HVRI and HVRII diversities in the 81 individuals from El Jadida led to the identification of the haplotypes reported in Table 1. The HVRI mtDNA diversity observed (Table 2) was, in general, as high as observed in other North African populations [4-6] and the Fu's Fs values for the neutrality tests were significantly negative, in accordance with populations in expansion, except, notoriously for the Libyan Tuaregs reported by [38].

**Table 1:** Haplotypes (for HVRI, HVRII and the four coding segments typed in possible H samples) and haplogroup classification in El Jadida.

Sample	HVRI	HVRII	Haplogroup	Other polymorphisms
J1	0	263 309.1 315.1	H*	

J2	129 184	146 263 309.2 315.1	H*	

J3	304	263 309.1 315.1	H*	

J4	0	263 315.1	H1	3010

J5	0	263 309.1 315.1	H1	3010

J6	0	263 309.2 315.1	H1	3010

J7	0	263 309.1 315.1	H1	3010

J8	0	263 309.2 315.1	H1	3010

J9	209	114 146 263 309.1 315.1	H1	3010

J10	212	263 309.1 315.1	H1	3010

J11	218	263 309.2 315.1	H1	3010

J12	318A/C	114 263 309.2 315.1	H1	3010

J13	355	263 315.1	H1	3010

J14	0	263 309.1 315.1	H7	4793

J15	67	263 309.1 315.1	HV1	

J16	93 298 311	72 263 315.1	V	

J17	153 193 298	72 195 263 309.1 315.1	V	

J18	153 298	72 195 263 315.1	V	

J19	193	72 93 263 309.1 315.1	V	

J20	298	72 195 263 309.1 315.1	V	

J21	298	72 263 309.1 315.1	V	

J22	298	72 263 309.1 315.1	V	

J23	298	72 195 263 309.2 315.1	V	

J24	126 291 362	58 64 152 263 315.1	R0a	7028

J25	126 291 362	58 64 152 263 315.1	R0a	7028

J26	126 362	58 64 263 315.1	R0a	

J27	93 224 311	73 263 309.1 315.1	K	

J28	224 287 311	73 146 263 309.1 315.1	K	

J29	224 287 311	73 146 263 309.1 315.1	K	

J30	224 287 311	73 146 263 309.1 315.1	K	

J31	224 287 311	73 146 263 309.1 315.1	K	

J32	224 287 311	73 146 263 309.1 315.1	K	

J33	224 311	73 263 280G/C 315.1	K	

J34	224 311	73 263 280C/G 315.1	K	

J35	69 126	73 185 228 263 295 315.1	J	

J36	69 126	73 185 225 228 263 295 315.1	J	

J37	69 126 193 300 309	73 263 309.1 315.1	J	

J38	69 126 193 265A/T	73 146 150 152 263 295 315.1	J2	

J39	69 126 193 278 291	73 150 152 263 295 309.1 315.1	J2	

J40	69 126 193 195 278	73 150 152 196insT 263 295 309.1 315.1	J2	

J41	126 220 292 294	73 146 152 195 263 279 315.1	T	

J42	126 163 186 189 193del 294	73 263 309.1 315.1	T1	

J43	173 183A/C 189 223 278	73 146 153 195 225 226 263 309.1 315.1	X	

J44	145 176C/G 223 311 390	73 152 204 263 315.1	N1b	

J45	0	73 195 263 315.1	U	7028

J46	0	73 195 263 315.1	U	7028

J47	311	263 309.1 315.1	U	7028

J48	287 356	73 195 263 309.1 315.1	U4	

J49	189 270	73 146 195 263 315.1	U5b	

J50	172 189 219 261 278	73 263 309.1 315.1	U6a	

J51	172 219 278 300	73 242 263 309.1 315.1	U6a	

J52	129 183A/C 189 223 235 249 311	73 152 195 263 315.3	M1	

J53	183A/C 189 249 265A/C 280 311	73 146 195 263 315.1	M1	

J54	183A/C 189 249 265A/C 280 311	73 146 195 263 315.1	M1	

J55	129 189 223 249 311 359	263 309.1 309.2 315.1	M1a	

J56	129 189 223 249 311 359	73 195 198 263 315.1	M1a	

J57	126 187 189 215A/T 223 264 270 278 293 311	73 152 182 185G/C 195 247 263 309.1 315.1 357	L1b1	

J58	126 187 189 223 264 270 278 293 311	73 152 182 185G/T 189 195 207 247 263 309.1 315.1 357	L1b1	

J59	126 187 189 223 264 270 278 293 311	73 152 182 185G/C 195 247 263 315.1 357	L1b1	

J60	126 187 189 223 264 270 278 293 311 355	73 152 182 185G/T 195 247 263 315.1 357	L1b1	

J61	126 187 189 223 264 270 278 293 311 355	73 152 182 185G/T 195 247 263 315.1 357	L1b1	

J62	17 148 163 187 189 223 278 293 294 311 360	73 89 93 151 182 186C/A 189A/C 247 263 315.1 316	L1c4	

J63	86 223 278 294 309 390	73 143 146 152 195 198 263 315.1	L2a1	

J64	167 192 223 278 294 309 390	73 143 146 152 195 263 309.1 315.1	L2a1	

J65	145 213 223 278 294 390	73 146 152 195 263 315.1	L2a1	

J66	189 192 223 278 294 309 357 390	73 143 146 152 195 263 315.1	L2a1	

J67	189 192 223 278 294 309	73 143 146 152 195 263 315.1	L2a1	

J68	51 172 223 266 278 362	73 263 315.1	L2c1	

J69	129 183A/C 189 278 300 354 357 390	73 146 150 195 263 309.2 315.1	L2d1	

J70	209 223	73 152 235 263 309.1 315.1	L3f1a	

J71	124 223 278 362	73 263 309.1 315.1	L3b1	

J72	124 223 336	73 152 242 263 315.1	L3d	

J73	124 223 256	73 152 189 195 263 315.1	L3d1'2'3'	

J74	124 192 223 256	73 152 189 195 263 309.1 315.1	L3d1'2'3'	

J75	223 320 399	73 152 195 198 263 315.1	L3e2a	

J76	223 311 320	73 150 195 198 263 315.1	L3e2a	

J77	172 183A/C 187 189 223 320	73 150 195 236 263 309.1 315.1 316	L3e2b	

J78	172 209 223 292 311	73 189 200 263 315.1	L3f1b	

J79	188 223 292 295 311	73 189 200 263 309.1 315.1	L3f1	

J80	209 223 292 311 390	73 189 200 263 315.1	L3f1b	

J81	129 223 256C/A 278 311 362	73 151 152 189A/C 195 263 294 309.1 315.1	L3h1b	

Variant positions from the rCRS are shown between 16017 and 16399 in HVRI (minus 16000), 72 and 357 for HVRII and the four coding region fragments (3001-3360, 3661-4050, 4281-4820, and 6761-7050). Substitutions are transitions unless the base change or a deletion is explicitly indicated. Insertions of one and two cytosines are shown by appending '1' and '2', respectively.

**Table 2:** Diversity measures in El Jadida and neighbour populations, within HVRI.

Sample	n	Different sequences (%)	Haplotype Diversity (SE)	Nucleotide Diversity (SE)	Mean no. pairwise differences (SE)	Tajima's D (p value)	Fu's Fs (p value)
El Jadida	81	59 (73%)	0.982 (0.008)	0.017 (0.009)	5.945 (2.866)	**-1.777 (0.007)**	**-25.241 (0.000)**

Algeria	86	31 (36%)	0.945 (0.010)	0.013 (0.007)	4.822 (2.377)	-1.090 (0.129)	**-13.198 (0.002)**

Morocco Berber	60	38 (63%)	0.963 (0.015)	0.013 (0.007)	4.594 (2.287)	**-1.862 (0.012)**	**-25.683 (0.000)**

Morocco non-Berber	32	29 (91%)	0.988 (0.014)	0.017 (0.009)	6.026 (2.948)	**-1.735 (0.022)**	**-24.805 (0.000)**

Souss	50	34 (68%)	0.961 (0.018)	0.013 (0.007)	4.604 (2.298)	**-1.551 (0.034)**	**-25.618 (0.000)**

Testour	50	36 (72%)	0.958 (0.021)	0.016 (0.009)	5.783 (2.814)	**-1.867 (0.008)**	**-25.269 (0.000)**

Slouguia	28	20 (71)	0.971 (0.018)	0.015 (0.008)	5.254 (2.618)	-1.472 (0.055)	**-10.159 (0.000)**

El Alia	48	27 (56%)	0.960 (0.016)	0.015 (0.008)	5.507 (2.695)	-1.385 (0.060)	**-12.910 (0.000)**

Qalaat El Andalous	29	17 (59%)	0.946 (0.024)	0.012 (0.007)	4.493 (2.278)	-0.946 (0.180)	**-6.559 (0.003)**

Tunis	51	44 (86%)	0.992 (0.006)	0.018 (0.010)	6.512 (3.131)	**-1.717 (0.024)**	**-25.154 (0.000)**

Zriba	35	17 (49%)	0.926 (0.028)	0.015 (0.008)	5.398 (2.665)	-1.156 (0.130)	-3.863 (0.076)

Kesra	43	30 (70%)	0.960 (0.020)	0.018 (0.010)	6.405 (3.094)	**-1.611 (0.027)**	**-17.708 (0.000)**

Skira	20	14 (70%)	0.937 (0.043)	0.011 (0.007)	4.137 (2.148)	**-1.598 (0.045)**	**-6.234 (0.003)**

Egypt	68	59 (87%)	0.993 (0.005)	0.020 (0.010)	7.075 (3.362)	**-1.768 (0.016)**	**-25.013 (0.000)**

Ethiopia	89	69 (78%)	0.990 (0.005)	0.023 (0.012)	8.145 (3.815)	**-1.705 (0.018)**	**-24.759 (0.000)**

Mauritania	30	23 (77%)	0.975 (0.017)	0.017 (0.009)	6.025 (2.953)	-0.738 (0.255)	**-12.983 (0.000)**

Nubia	80	53 (66%)	0.977 (0.008)	0.023 (0.012)	8.203 (3.844)	-1.420 (0.056)	**-24.779 (0.000)**

Senegal	50	42 (84%)	0.989 (0.008)	0.017 (0.009)	6.283 (3.032)	-1.127 (0.141)	**-25.208 (0.000)**

Serer	23	21 (91%)	0.992 (0.015)	0.023 (0.012)	8.340 (4.009)	-1.036 (0.152)	**-11.679 (0.000)**

Libya	129	20 (16%)	0.677 (0.046)	0.011 (0.006)	3.983 (2.005)	-1.254 (0.088)	-2.726 (0.223)

SE stands for standard error. Values in bold are statistically significant at the 5% level.

The analysis of molecular variance (AMOVA) was performed in order to evaluate genetic structure within North Africa, revealing a residual 3% variation between populations. Relatively to pairwise F_ST _genetic distances (not shown), the only significant values after Bonferroni's correction were between El Jadida-Algeria (0.061; p value = 0.000 ± 0.000), El Jadida-Tuaregs from Libya (0.022; p = 0.000 ± 0.000), El Jadida-Morocco-Berbers (0.027; p = 0.000 ± 0.000) and El Jadida-El Alia from Tunisia (0.024; p = 0.000 ± 0.000).

When analyzing the proportions of sub-Saharan and West Eurasian mtDNA haplogroups (Table 1) in El Jadida population, the characteristic mixed pool was observed, with frequencies of 30.86% and 69.14%, respectively. The sub-Saharan pool presented the branches L1, L2 and L3, in the following frequencies: 24%, 28% and 48% of the sub-Saharan pool. The basal haplogroup L0 was absent. In the West Eurasian pool, the haplogroups said to have been introduced into North and East Africa as result of a Back-to-Africa migration from the Near East, U6 and M1, were observed with frequencies of 2.47% and 6.17% in El Jadida.

Clearly, the main component of the West Eurasian lineages was made of possible Iberian expanded lineages following the post-glacial climate improvement: H1 (12.35%), V (9.88%) and U5b (1.23%). There were low frequent lineages belonging to the HV branch of the maternal tree which could have come to El Jadida from the Near East, (H* - 3.70%; H7 - 1.23%; HV1 - 1.23%) as well as R0a (3.70%), X (1.23%), N1b (1.23%), J (7.41%), T (2.47%). There was also a considerable amount of U/K lineages, besides the already referred U6 and U5a: K (9.88%), U* (3.70%) and U4 (1.23%). Curiously, five out of eight K individuals in El Jadida presented a substitution on position 16287 (besides the haplogroup defining 16224-16311 polymorphisms); this haplotype was so far observed in 1 Italian (belonging to sub-haplogroup K1a4) and two Moroccan individuals (sub-haplogroup K1a2) out of 789 K sequences in [2] and absent in other North African populations [6].

### Sub-Saharan haplogroups across North Africa

Based on a database summing up 4908 African and 2178 Near Eastern/Arabian Peninsula individuals (Figure 2A shows sample locations, further indicated in Additional File 1) we assayed interpolation analyses of L haplogroup frequencies. As can be seen in Figure 2B, the north to south increase of frequency across North Africa and the Sahara is visible. In the East of the African continent, the highest L frequencies are attained in more southern latitudes than in the rest of the continent, due to presence of M and some N (R0a and U6) lineages, especially high in Ethiopia.

We then focused attention in the region across Sahara, for each of the main L haplogroups. When interpolation analyses are performed for the frequencies in total population, any sign of gradient across the Sahara is lost, as differences between L frequencies southern and northern of the desert are high. For this reason, interpolation analyses were performed for the frequencies of each haplogroup in the L pool, enhancing the possibility of detecting gradients across the Sahara.

**Figure 3:**
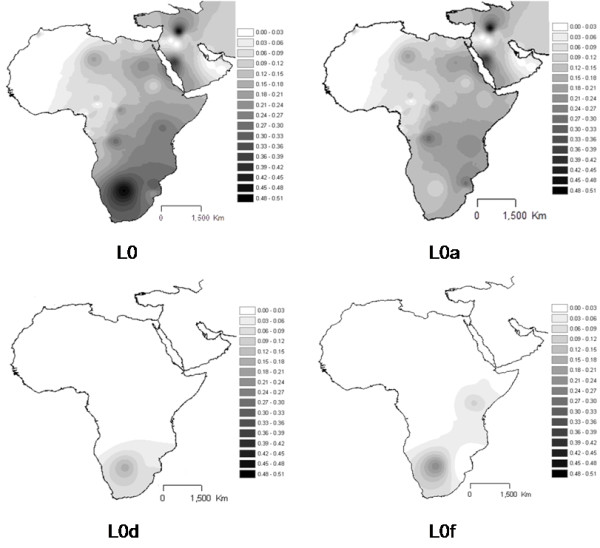
**Interpolation maps for L0 haplogroup in the sub-Saharan pool observed in each sample**.

L0 (Figure 3) attains the higher proportion inside L pool in East Africa, including the Near East and Arabian Peninsula, following a decreasing frequency from south towards north. This pattern is coincident with the one for haplogroup L0a, while L0d and L0f are almost restricted to the south.

**Figure 4:**
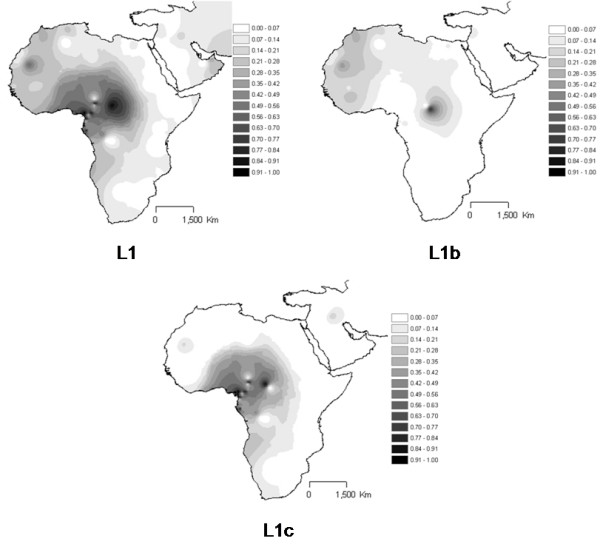
**Interpolation maps for L1 haplogroup in the sub-Saharan pool observed in each sample**.

L1 total (Figure 4) attains the highest proportions in the L pool in central Africa, in Pygmy populations, followed by some of the north-west populations. This presence of L1 in north-west African samples is mainly due to L1b sub-haplogroup, while L1c is quite restricted to Central Africa. The presence of this haplogroup in Near East and Arabian Peninsula is quite limited.

**Figure 5:**
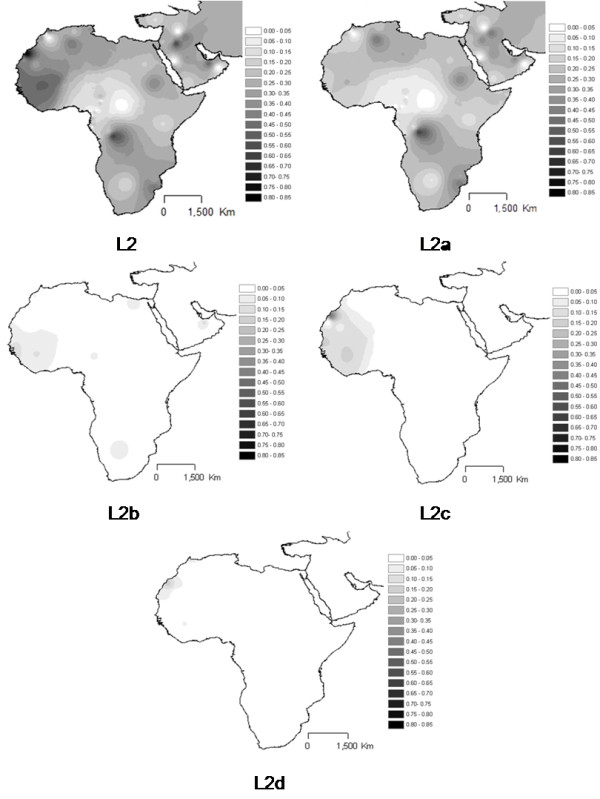
**Interpolation maps for L2 haplogroup in the sub-Saharan pool observed in each sample**.

L2 total (Figure 5) is one of the two dominant haplogroups in the L pool, in many regions across Africa, namely in central-west and south-east regions, most probably due to Bantu expansion [11,12] and towards north-west, potentially due to the trans-Saharan slave trade. The very central African populations, mostly Pygmy groups, present low proportions of L2 lineages in its pool. This pattern is caused mainly by sub-haplogroup L2a, the most frequent lineage in L2, while L2b, L2c and L2d attain highest proportions in the west coast between Senegal and Mauritania.

**Figure 6:**
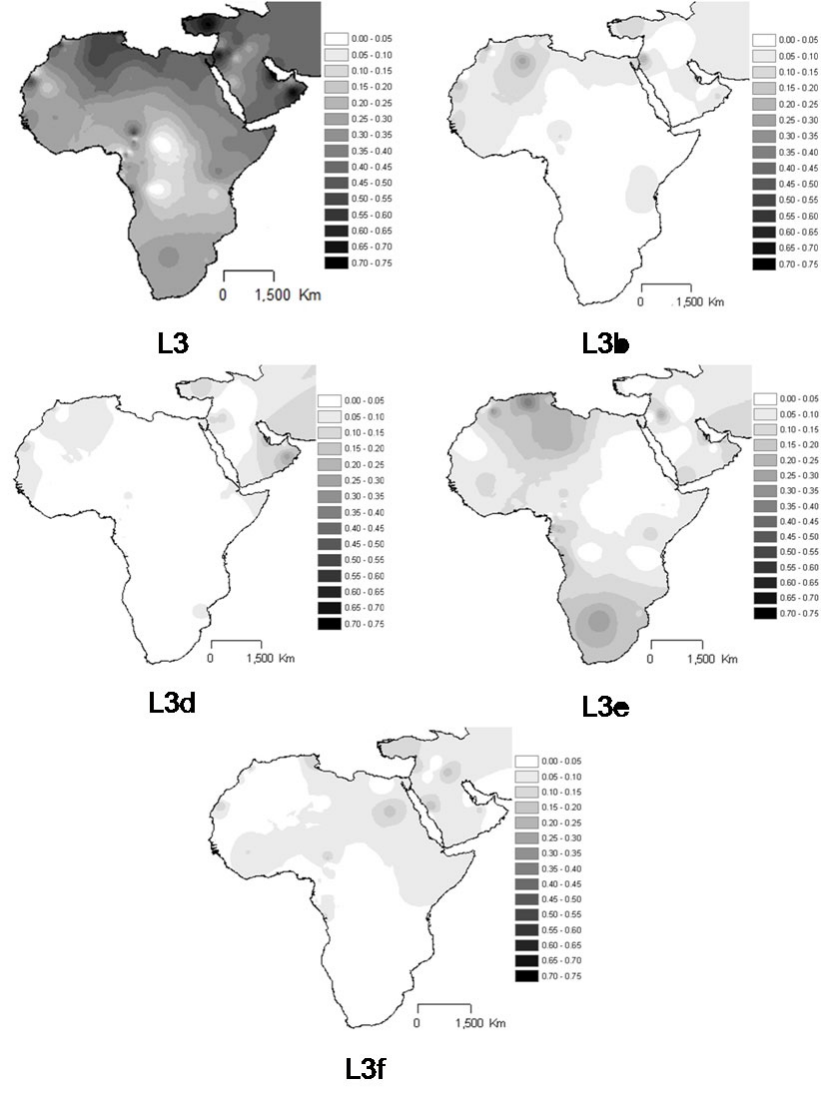
**Interpolation maps for L3 total, L3b, L3d, L3e and L3f haplogroups in the sub-Saharan pool observed in each sample**.

**Figure 7:**
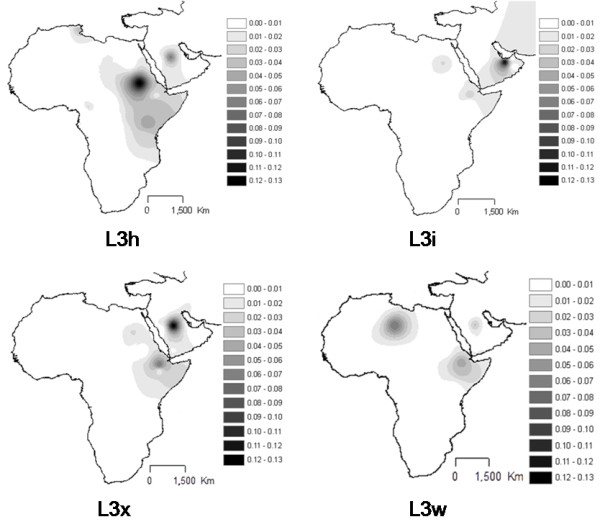
**Interpolation maps for L3h, L3i, L3x and L3w haplogroups in the sub-Saharan pool observed in each sample**.

L3 total (Figure 6) reaches the highest proportions in North and then east Africa. The sub-haplogroups L3b and L3d clearly dominate in the west, as known before, as well as in North Africa. L3e has a more central dispersion across Sahara, being also frequent in South Africa. L3f has an eastern localization across the Sahara, with some foci in Central Africa southern of Sahara, due to high frequencies of L3f3 in Chadic-speaking groups [1]. L3h, L3i, L3w and L3x (Figure 7) are rare and clearly limited to East Africa.

**Figure 8:**
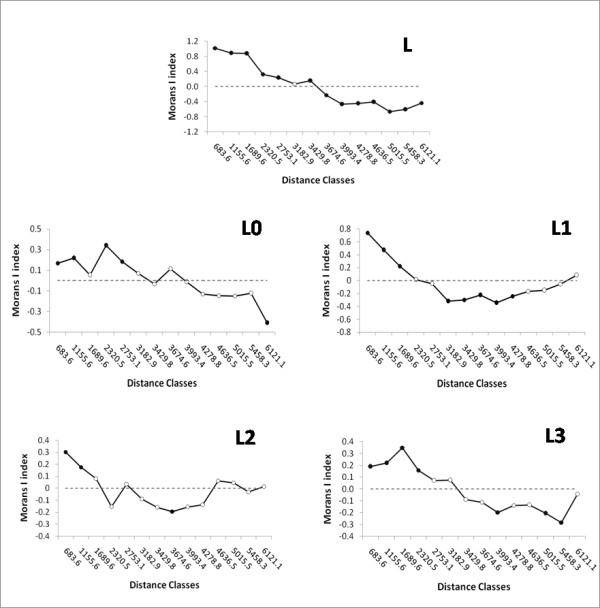
**Spatial correlograms of Moran's I indeces for the total L frequency in the populations, and for the L0, L1, L2 and L3 proportions of the sub-Saharan pools in the samples**. Geographic distances separating samples are distributed into 14 classes. Full dots represent significant p-values (p < 0.05); empty dots are non-significant p-values.

When the spatial autocorrelation analysis was applied to the total L frequency in the populations, and to the L0, L1, L2 and L3 proportions of the sub-Saharan pools in the samples, signs of cline were evident for all them (Figure 8). The positive values at small distances indicate that individuals from the same population are more similar to each other; while the negative values at the largest distances (not so clear for L1 and L2) suggest a marked genetic differentiation across the African continent and Arabian Peninsula.

### Complete L3 sequences

**Figure 9:**
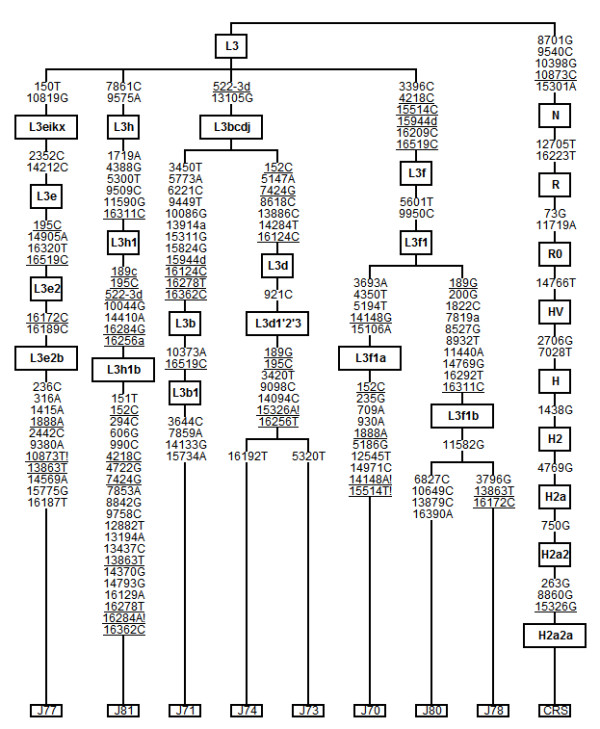
**Phylogeny of the complete L3 sequences from El Jadida**. Integers represent transitions when the suffixes "A", "G", "C" or "T" are appended and transversions when the suffixes "a", "g", "c" or "t" are appended. Deletions are indicated by a "d" following the deleted nucleotide position. Underlined nucleotide positions appear more than once in the tree.

We performed the complete sequencing of 8 L3 different haplotypes observed in El Jadida. This haplogroup was selected because it is the most diversified sub-Saharan haplogroup in El Jadida and some of its lineages could have been inputted in North Africa from East Africa. The complete sequencing allowed the fine characterization of these samples as follows (Figure 9): one L3b1, two L3d1'2'3, one L3e2b, one L3f1a, two L3f1b and one L3h1b.

Joining these 8 complete L3 sequences to 236 previously published ones (the ones summed up in [1,14,20-22]), a good resolution of L3(xM, N) tree is obtained (Additional File 2; information for samples used is listed in Additional File 3). There are 39 sequences from North Africa, representing 16% of the complete L3 dataset, being 10 from Morocco, one from Algeria, four from Libya, 11 from Tunisia, and 13 from Egypt. So this work raised the homogeneity of complete L3 sequences across North Africa.

Most of these North African sequences share a recent ancestry with sequences observed in other parts of Africa, in the Holocene period (Table 3). This seems to point to a recent introduction of these lineages in North Africa from the original locations in sub-Saharan and East Africa. Namely, one Moroccan and one Libyan sequences belong to sub-haplogroup L3b1b, together with two West African sequences from Burkina, with a coalescence age of 9,926 ± 2,555 years. Three Egyptian, four Tunisian, one Libyan and one Moroccan sequences share a most recent common ancestor of 13,537 ± 1,058 years old with seven West African, two South African, six Americans (most probably African-descents), two East Africans, two Central Africans, five Near Eastern and two South Asians, being affiliated in haplogroup L3b1a. A Moroccan sequence shares an ancestry with one sequence from Guinea-Bissau of around 13,370 ± 4,205 years old, inside haplogroup L3b2. One Tunisian L3d1c sequences share an ancestor with one American African-descent at 9,246 ± 3,444 years ago. One Tunisian shares an ancestor at around 6,549 ± 2,883 years ago with one Syrian inside L3d1'2'3 haplogroup. One Tunisian and one Egyptian together with four individuals from Burkina, one from Guinea Bissau and two Americans share an ancestor at 14,179 ± 2,352 years ago, belonging to the haplogroup L3e2a. In haplogroup L3e2b, two Egyptians and one Moroccan share a most recent common ancestor at 11,985 ± 1,529 years ago with one Ethiopian, one Zaire, three West Africans and five Americans (with an younger co-ancestry between the Egyptian and one American at around 1,287 ± 1,278 years ago inside L3e2b2). One Egyptian, one Libyan and one Tunisian L3e5 sequences share an ancestor of 11,516 ± 2,264 years with one Burkina, one Ethiopian, one Sudanese and one American (with a somewhat younger co-ancestry between the Tunisian and the Ethiopian at around 10,610 ± 3,704 years ago). A Moroccan L3f1a shares a common ancestor with one Chadic sample at 14,766 ± 4,448 years ago. L3f1b haplogroup, having a most recent common ancestor of 14,710 ± 1,227 years old, bears some sequences from North Africa (two Egyptians and two Moroccan), and many other from other African locations and Near Eastern, with one Egyptian sample having an younger co-ancestor, at 4,343 ± 2,388 years ago, with one Jordanian and one American.

**Table 3:** Age estimates and standard deviations (in years) for the Most Recent Common Ancestor for the related lineages in North and sub-Saharan Africa.

Clade	Related lineages	Age ± standard deviation (years)
L3b1b	4-7	9,926 ± 2,555

L3b1a	8-39, 229, 230, 233	13,537 ± 1,058

L3b2	42, 234	13,370 ± 4,205

L3d1c	61, 62	9,246 ± 3,444

L3d1'2'3	76, 231	6,549 ± 2,883

L3e2a	107-115	14,179 ± 2,352

L3e2b	116-129, 235, J77	11,985 ± 1,529

L3e2b2	128, 129	1,287 ± 1,278

L3e5	144-148, 232	11,516 ± 2,264

L3e5	147, 232	10,610 ± 3,704

L3f1a	165, J70	14,766 ± 4,448

L3f1b	167-189, 214, 236, J78, J80	14,710 ± 1,227

L3f1b2	178, 179, 236	4,343 ± 2,388

L3f2b	192, 193	24,809 ± 5,935

L3h1a2	199, 200	26,281 ± 6,139

L3h1b	204-210, J81	36,827 ± 3,772

L3h1b	204, J81	14,766 ± 4,448

L3 × 2	155-159	33,165 ± 4,499

Numbers in the "related lineages" column refer to identification of samples in the tree in Additional File 2.

A few L3 sequences observed in North Africa have older co-ancestry with other sub-Saharan regions, but as this occurs in the rarer haplogroups (almost restricted to East Africa), most probably the scenario will change as these become better characterized. This is the case for one L3 × 2 sequence observed in Algeria, which shares an older most recent common ancestor with two Ethiopian, one Israeli and one Kuwait, at 33,165 ± 4,499 years ago, but one Ethiopian and the Israeli and Kuwait sequences share a younger ancestor at 19,012 ± 4,200. Also, one Egyptian L3f2b sequence shares an ancestor with a Chadic one at around 24,809 ± 5,935 years ago. For L3 h1a2 haplogroup, one Egyptian and one Lebanese sequences share a coalescence age of 26,281 ± 6,139 years old. And for L3 h1b, with an age of 36,827 ± 3,772 years, one of the North African sequences (one Tunisian and one Moroccan) has a most recent common ancestor of 14,766 ± 4,448 years old with a sequence from Guinea Bissau.

So far, the two only complete published samples belonging to haplogroup L3k have a North African origin, one from Libya and one from Tunisia. This haplogroup has a coalescent age of around 29,251 ± 6,524 years old. As it is impossible to identify this haplogroup based only in control region information (only through HVRII polymorphism at position 235), it is impossible to add additional information about this haplogroup.

## Conclusions

The genetic information testifies that recent migrations were the main events leading to the mtDNA pool observed nowadays in Maghreb populations. The ancestral Near Eastern pool, remnant of the ancient Back-to-Africa migration through the Levant around 40,000 years ago [9] is very restricted. Values for these haplogroups are around 8.6% in El Jadida and 10% in Tunisia [6]. A bulk of the West Eurasian lineages present in Maghreb populations is constituted by the typical Iberian sub-haplogroups H and V (12.3% and 9.9%, respectively, in El Jadida). It is highly probable that these lineages did expand towards North Africa when they expanded to the rest of the European continent, from Iberia, around 14,000 years ago, as they are present in all North African populations, even in those not known as directly historically related with Iberia [6].

Recent mtDNA data have shown that considerable local population expansions occurred in Sahel nomadic populations around 4,000 years ago, following important movements of northern and eastern African people towards the recently formed Sahel region. These local expansions were revealed in one branch of the typical East African haplogroups L3f, the L3f3 almost restricted to the Chadic-speaking nomadic groups [1] and in one branch of the typical Iberian haplogroup V in southern Tuareg populations [8]. Thus, the emergence of the modern Sahara, beginning some 4,000 years ago, hardened existing geographical divisions and separated peoples, forcing the black Saharans into the oases or southwards into the more attractive lands of the Sahel.

This barrier in gene flow is evident when attending to the global L haplogroup frequencies in African populations. There is a clear horizontal gradient across the continent, attaining values of 95% and higher in the Sahel region in West and Central Africa, but not in the Eastern African coast where those values are only reached around the border between Tanzania and Mozambique. The lower values for L frequencies in the eastern African coast are due to the southern migration of the Eurasian haplogroup M1, which is typical of East Africa. North Africa reaches L frequencies of 20-40%, while the Arabian Peninsula and the Near East have around 20-30% (only higher in Yemen).

The coalescence ages for the L sequences observed nowadays in North Africa shows the young ancestry of these lineages, which were originated in sub-Saharan Africa in the Holocene. This proves that sub-Saharan people did not leave traces in the maternal gene pool for the time of settlement of North Africa, some 40,000 years ago. And for sure, the continuous publishing of complete L sequences across Africa will reveal still younger ancestors between L sequences observed in both sides of the Saharan desert, bringing its introduction into North Africa to more recent/historical times.

It is also relevant that the interpolation analyses of haplogroups inside the L pool across the Sahara revealed horizontal gradients, matching in a high extent the known trans-Saharan routes. The West is dominated by L1b, L2b, L2c, L2d, L3b and L3d. The Center has L3e and some L3f and L3w. The East bears L0a, L3h, L3i, L3x and, in common with the Center, L3f and L3w. L2a is almost everywhere, strengthening its dominance in the slave package, not only towards the New World, but also in the trans-Saharan trade.

Both these genetic evidences agree with historical data that the introduction of the Asiatic horse into North Africa around 2,000 years ago lengthened the reach of desert nomads' raiding and trading. Before this period, the few black slaves taken from time to time across the Sahara would have been seen on the far side of the Mediterranean as mere exotic household ornaments. But, it may be argued that there was no regular trans-Saharan trade system before the rise of the camel-mounted Berber nomad, in the first Christian centuries, and perhaps not even until after the arrival of the first camel-riding Muslim Arabs in North Africa, in the seventh century [39].

## Authors' contributions

NH, MDC and MK carried out the molecular genetic studies. NH, MDC, VF, MK and JBP conducted the sequence alignment and editing, assigned sequences to haplogroups, estimated ages for lineages and performed the general statistical analyses for evaluation of genetic diversity. NMS MDC and VF performed the interpolation analyses. LP designed the study, supervised the work and drafted the manuscript in collaboration with the other authors. All authors read and approved the final manuscript.

## Supplementary Material

Additional file 1 **Information for samples used in the interpolation analyses**. Information about size, ethnic group, location and bibliographic reference for samples used in the interpolation analyses. Click here for file

Additional file 2 **Phylogeny of complete L3 sequences**. Phylogenetic tree reconstruction for 244 complete L3 sequences. Integers represent transitions when the suffixes "A", "G", "C" or "T" are appended and transversions when the suffixes "a", "g", "c" or "t" are appended. Deletions are indicated by a "d" following the deleted nucleotide position. Underlined nucleotide positions appear more than once in the tree. TMRCAs are represented inside boxes. Click here for file

Additional file 3 **Information for samples used in the phylogeny of complete L3 sequences**. Information about location, bibliographic reference and GenBank Accession Number for samples used in the phylogeny of complete L3 sequences. Click here for file
