# Supplementary material for: The trans-Saharan slave trade - clues from interpolation analyses and high-resolution characterization of mitochondrial DNA lineages
Source: BMC Evol Biol. 2010 May 10;10:138. doi: 10.1186/1471-2148-10-138 (PMC2875235; doi:10.1186/1471-2148-10-138)
Supplement: Additional file 1 — Information for samples used in the interpolation analyses. Information about size, ethnic group, location and bibliographic reference for samples used in the interpolation analyses. [file 1471-2148-10-138-S1.DOC]

Supplementary material 1 – Information for samples used in the interpolation analyses.

| **Sample code** | **Location (ethnic group)** | **Sample size** | **References** |
| --- | --- | --- | --- |
| ETK | Ethiopia | 270 | Kivisild et al. (2004) |
| ANP | Angola | 44 | Plaza et al. (2004) |
| BIW | Central African Republic (Biaka Pygmies) | 17 | Watson et al. (1997) |
| KEB | Kenya | 100 | Branstatter et al. (2004) |
| ANB | Angola | 109 | Beleza et al. (2005) |
| CNC | Cameroon north | 244 | Coia et al. (2005) |
| CSC | Cameroon south | 197 | Coia et al. (2005) |
| EGM | Equatorial Guinea | 95 | Mateu et al. (1997) |
| CNH | Cameroon north | 104 | Cerný et al. (2004) |
| SAC | South Africa (Khwe) | 31 | Chen et al. (2000) |
| MAE | Mali | 61 | Ely et al. (2006) |
| BAE | Mali | 19 | Ely et al. (2006) |
| MBW | Zaire (Mbuti Pygmies) | 20 | Watson et al. (1997) |
| MOS | Mozambique | 307 | Salas et al. (2002) |
| MOL | Mozambique | 109 | Pereira et al. (2001) |
| NUK | Nubia | 80 | Krings et al. (1999) |
| CCD | Cameroon and Central African Republic | 158 | Destro-Bisol et al. (2004) |
| SUK | Sudan | 76 | Krings et al. (1999) |
| TAK | Tanzania | 100 | Knight et al. (2003) |
| KEW | Kenya (Kikuyu; Somali; Turkana) | 88 | Watson et al. (1997) |
| GWQ | Gabon west | 159 | Quintana-Murci et al. (2008) |
| GCQ | Gabon center | 147 | Quintana-Murci et al. (2008) |
| GEQ | Gabon east | 154 | Quintana-Murci et al. (2008) |
| GNQ | Gabon north and northwest | 116 | Quintana-Murci et al. (2008) |
| GSQ | Gabon southeast | 255 | Quintana-Murci et al. (2008) |
| CWQ | Cameroon west | 152 | Quintana-Murci et al. (2008) |
| GPQ | Gabon northeast and southeast (Pygmies) | 115 | Quintana-Murci et al. (2008) |
| BIQ | Central African Republic (Biaka Pygmies) | 56 | Quintana-Murci et al. (2008) |
| MBQ | Democratic Republic of Congo (Mbuti Pygmies) | 39 | Quintana-Murci et al. (2008) |
| CCQ | Cameroon center (Pygmies) | 30 | Quintana-Murci et al. (2008) |
| CSQ | Cameroon southwest (Pygmies) | 58 | Quintana-Murci et al. (2008) |
| CPQ | Cameroon west (Pygmies) | 88 | Quintana-Murci et al. (2008) |
| CNQ | Cameroon north (Pygmies) | 35 | Quintana-Murci et al. (2008) |
| MOC | Algeria (Mozabite) | 86 | Côrte-Real et al. (1996) |
| ALP | Algeria | 47 | Plaza et al. (2003) |
| EGK | Egypt | 68 | Krings et al. (1999) |
| MOR | Morocco | 32 | Rando et al. (1998) |
| MOB | Morocco | 50 | Brakez et al. (2001) |
| MOP | Morocco | 18 | Plaza et al. (2003) |
| MBR | Morocco (Berbers) | 60 | Rando et al. (1998) |
| TUP | Tunisia | 47 | Plaza et al. (2003) |
| JAD | Morocco | 81 | This work |
| LIO | Libya (Tuaregs) | 129 | Ottoni et al. (2009) |
| TUC | Tunisia | 304 | Cherni et al. (2009) |
| MAR | Mauritania | 30 | Rando et al. (1998) |
| SAR | West Sahara | 25 | Rando et al. (1998) |
| SAP | West Sahara | 56 | Plaza et al. (2003) |
| WOR | Senegal (Wolof) | 48 | Rando et al. (1998) |
| SER | Senegal (Serer) | 23 | Rando et al. (1998) |
| SEM | Senegal | 50 | Rando et al. (1998) |
| MAG | Senegal (Mandenka) | 119 | Graven et al. (1995) |
| CAA | Central Arabia | 289 | Abu Amero et al. (2007) |
| NAA | North Arabia | 43 | Abu Amero et al. (2007) |
| SAA | South-Eastern Arabia | 113 | Abu Amero et al. (2007) |
| WAA | Western Arabia | 72 | Abu Amero et al. (2007) |
| YEM | Yemen | 350 | Cerný et al. (2008); Kivisild et al. (2004); Rowold et al. (2007) |
| OMR | Oman | 105 | Rowold et al. (2007) |
| QAR | Qatar | 90 | Rowold et al. (2007) |
| UAE | United Arab Emirates | 131 | Rowold et al. (2007) |
| BED | Jeddah (Beduins) | 29 | Di Rienzo and Wilson (1991) |
| IRR | Iraq | 117 | Richards et al. (2000) |
| SYR | Syria | 69 | Richards et al. (2000) |
| PAR | Palestina | 109 | Richards et al. (2000) |
| JOR | Jordan | 185 | González et al. (2008); Rowold et al. (2007) |
| TUK | Turkey | 174 | Calafell et al. (1996); Comas et al. (1996); DiBenedetto et al. (2001); Richards et al. (1996) |
| KUR | Kurdistan | 53 | Richards et al. (2000) |
| DUA | Dubai | 249 | Ashamali et al. (2008) |

Abu-Amero KK, González AM, Larruga JM, Bosley TM, Cabrera VM. 2007. [Eurasian and African mitochondrial DNA influences in the Saudi Arabian population.](http://www.ncbi.nlm.nih.gov/pubmed/17331239?ordinalpos=15&itool=EntrezSystem2.PEntrez.Pubmed.Pubmed_ResultsPanel.Pubmed_DefaultReportPanel.Pubmed_RVDocSum) BMC Evol Biol.7:32

Alshamali F, Brandstätter A, Zimmermann B, Parson W. 2008. Mitochondrial DNA control region variation in Dubai, United Arab Emirates. Forensic Sci Int Genet: Genetics 2:e9-e10.

Beleza S, Gusmão L, Amorim A, Carracedo A, Salas A. 2005. The genetic legacy of western Bantu migrations. Hum Genet 117: 366–375.

[Brakez Z](http://www.ncbi.nlm.nih.gov/sites/entrez?Db=pubmed&Cmd=Search&Term="Brakez Z"%5BAuthor%5D&itool=EntrezSystem2.PEntrez.Pubmed.Pubmed_ResultsPanel.Pubmed_DiscoveryPanel.Pubmed_RVAbstractPlus), [Bosch E](http://www.ncbi.nlm.nih.gov/sites/entrez?Db=pubmed&Cmd=Search&Term="Bosch E"%5BAuthor%5D&itool=EntrezSystem2.PEntrez.Pubmed.Pubmed_ResultsPanel.Pubmed_DiscoveryPanel.Pubmed_RVAbstractPlus), [Izaabel H](http://www.ncbi.nlm.nih.gov/sites/entrez?Db=pubmed&Cmd=Search&Term="Izaabel H"%5BAuthor%5D&itool=EntrezSystem2.PEntrez.Pubmed.Pubmed_ResultsPanel.Pubmed_DiscoveryPanel.Pubmed_RVAbstractPlus), [Akhayat O](http://www.ncbi.nlm.nih.gov/sites/entrez?Db=pubmed&Cmd=Search&Term="Akhayat O"%5BAuthor%5D&itool=EntrezSystem2.PEntrez.Pubmed.Pubmed_ResultsPanel.Pubmed_DiscoveryPanel.Pubmed_RVAbstractPlus), [Comas D](http://www.ncbi.nlm.nih.gov/sites/entrez?Db=pubmed&Cmd=Search&Term="Comas D"%5BAuthor%5D&itool=EntrezSystem2.PEntrez.Pubmed.Pubmed_ResultsPanel.Pubmed_DiscoveryPanel.Pubmed_RVAbstractPlus), [Bertranpetit J](http://www.ncbi.nlm.nih.gov/sites/entrez?Db=pubmed&Cmd=Search&Term="Bertranpetit J"%5BAuthor%5D&itool=EntrezSystem2.PEntrez.Pubmed.Pubmed_ResultsPanel.Pubmed_DiscoveryPanel.Pubmed_RVAbstractPlus), [Calafell F](http://www.ncbi.nlm.nih.gov/sites/entrez?Db=pubmed&Cmd=Search&Term="Calafell F"%5BAuthor%5D&itool=EntrezSystem2.PEntrez.Pubmed.Pubmed_ResultsPanel.Pubmed_DiscoveryPanel.Pubmed_RVAbstractPlus). 2001. Human mitochondrial DNA sequence variation in the Moroccan population of the Souss area. [Ann Hum Biol.](javascript:AL_get(this, 'jour', 'Ann Hum Biol.');) 28:295-307.

Brandstätter A, Peterson CT, Irwin JA, Mpoke S, Koech DK, Parson W, Parsons TJ. 2004. Mitochondrial DNA control region sequences from Nairobi (Kenya): inferring phylogenetic parameters for the establishment of a forensic database. Int J Legal Med 118:294–306.

Calafell F, Underhill P, Tolun A, Angelicheva D, Kalaydjieva L. 1996. [From Asia to Europe: mitochondrial DNA sequence variability in Bulgarians and Turks.](http://www.ncbi.nlm.nih.gov/pubmed/8835097?ordinalpos=8&itool=EntrezSystem2.PEntrez.Pubmed.Pubmed_ResultsPanel.Pubmed_DefaultReportPanel.Pubmed_RVDocSum) Ann Hum Genet. 60:35-49.

Cerný V, Hájek M, Cmejla R, Brůzek J, Brdicka R. 2004. [mtDNA sequences of Chadic-speaking populations from northern Cameroon suggest their affinities with eastern Africa.](http://www.ncbi.nlm.nih.gov/pubmed/15739384?ordinalpos=7&itool=EntrezSystem2.PEntrez.Pubmed.Pubmed_ResultsPanel.Pubmed_DefaultReportPanel.Pubmed_RVDocSum) Ann Hum Biol. 31:554-69.

[Cerný V](http://www.ncbi.nlm.nih.gov/sites/entrez?Db=pubmed&Cmd=Search&Term="Cerný V"%5BAuthor%5D&itool=EntrezSystem2.PEntrez.Pubmed.Pubmed_ResultsPanel.Pubmed_DiscoveryPanel.Pubmed_RVAbstractPlus), [Mulligan CJ](http://www.ncbi.nlm.nih.gov/sites/entrez?Db=pubmed&Cmd=Search&Term="Mulligan CJ"%5BAuthor%5D&itool=EntrezSystem2.PEntrez.Pubmed.Pubmed_ResultsPanel.Pubmed_DiscoveryPanel.Pubmed_RVAbstractPlus), [Rídl J](http://www.ncbi.nlm.nih.gov/sites/entrez?Db=pubmed&Cmd=Search&Term="Rídl J"%5BAuthor%5D&itool=EntrezSystem2.PEntrez.Pubmed.Pubmed_ResultsPanel.Pubmed_DiscoveryPanel.Pubmed_RVAbstractPlus), [Zaloudková M](http://www.ncbi.nlm.nih.gov/sites/entrez?Db=pubmed&Cmd=Search&Term="Zaloudková M"%5BAuthor%5D&itool=EntrezSystem2.PEntrez.Pubmed.Pubmed_ResultsPanel.Pubmed_DiscoveryPanel.Pubmed_RVAbstractPlus), [Edens CM](http://www.ncbi.nlm.nih.gov/sites/entrez?Db=pubmed&Cmd=Search&Term="Edens CM"%5BAuthor%5D&itool=EntrezSystem2.PEntrez.Pubmed.Pubmed_ResultsPanel.Pubmed_DiscoveryPanel.Pubmed_RVAbstractPlus), [Hájek M](http://www.ncbi.nlm.nih.gov/sites/entrez?Db=pubmed&Cmd=Search&Term="Hájek M"%5BAuthor%5D&itool=EntrezSystem2.PEntrez.Pubmed.Pubmed_ResultsPanel.Pubmed_DiscoveryPanel.Pubmed_RVAbstractPlus), [Pereira L](http://www.ncbi.nlm.nih.gov/sites/entrez?Db=pubmed&Cmd=Search&Term="Pereira L"%5BAuthor%5D&itool=EntrezSystem2.PEntrez.Pubmed.Pubmed_ResultsPanel.Pubmed_DiscoveryPanel.Pubmed_RVAbstractPlus). 2008. Regional differences in the distribution of the sub-Saharan, West Eurasian, and South Asian mtDNA lineages in Yemen. [Am J Phys Anthropol.](javascript:AL_get(this, 'jour', 'Am J Phys Anthropol.');) 136:128-37.

Chen YS, Olckers A, Schurr TG, Kogelnik AM, Huoponen K, Wallace DC. 2000. [mtDNA variation in the South African Kung and Khwe-and their genetic relationships to other African populations.](http://www.ncbi.nlm.nih.gov/pubmed/10739760?ordinalpos=33&itool=EntrezSystem2.PEntrez.Pubmed.Pubmed_ResultsPanel.Pubmed_DefaultReportPanel.Pubmed_RVDocSum) Am J Hum Genet. 66:1362-83.

Cherni L, Fernandes V, Pereira JB, Costa MD, Goios A, Frigi S, Yacoubi-Loueslati B, Amor MB, Slama A, Amorim A, El Gaaied AB, Pereira L. 2009. [Post-last glacial maximum expansion from Iberia to North Africa revealed by fine characterization of mtDNA H haplogroup in Tunisia.](http://www.ncbi.nlm.nih.gov/pubmed/19090581?ordinalpos=4&itool=EntrezSystem2.PEntrez.Pubmed.Pubmed_ResultsPanel.Pubmed_DefaultReportPanel.Pubmed_RVDocSum) Am J Phys Anthropol. 139:253-60.

Coia V, Destro-Bisol G, Verginelli F, Battaggia C, Boschi I, Cruciani F, Spedini G, Comas D, Calafell F. 2005. Brief communication: mtDNA variation in North Cameroon: lack of Asian lineages and implications for back migration from Asia to sub-Saharan Africa. Am J Phys Anthropol. 128:678-81.

Comas D, Calafell F, Mateu E, Pérez-Lezaun A, Bertranpetit J. 1996. [Geographic variation in human mitochondrial DNA control region sequence: the population history of Turkey and its relationship to the European populations.](http://www.ncbi.nlm.nih.gov/pubmed/8865661?ordinalpos=1&itool=EntrezSystem2.PEntrez.Pubmed.Pubmed_ResultsPanel.Pubmed_DefaultReportPanel.Pubmed_RVDocSum) Mol Biol Evol. 13:1067-77.

Côrte-Real HB, Macaulay VA, Richards MB, Hariti G, Issad MS, Cambon-Thomsen A, Papiha S, Bertranpetit J, Sykes BC. 1996. [Genetic diversity in the Iberian Peninsula determined from mitochondrial sequence analysis.](http://www.ncbi.nlm.nih.gov/pubmed/8865993?ordinalpos=3&itool=EntrezSystem2.PEntrez.Pubmed.Pubmed_ResultsPanel.Pubmed_DefaultReportPanel.Pubmed_RVDocSum) Ann Hum Genet. 60:331-50.

Destro-Bisol G, Coia V, Boschi I, Verginelli F, Cagliá A, Pascali V, Spedini G, Calafell F. 2004. [The analysis of variation of mtDNA hypervariable region 1 suggests that Eastern and Western Pygmies diverged before the Bantu expansion.](http://www.ncbi.nlm.nih.gov/pubmed/14970923?ordinalpos=2&itool=EntrezSystem2.PEntrez.Pubmed.Pubmed_ResultsPanel.Pubmed_DefaultReportPanel.Pubmed_RVDocSum) Am Nat. 163:212-26.

Di Benedetto G, Erguven A, Stenico M, Castri L, Bertorelle G, Togan I, Barbujani G. 2001. DNA diversity and population admixture in Anatolia. Am J Phys Anthropol 115:144-156.

[Di Rienzo A](http://www.ncbi.nlm.nih.gov/sites/entrez?Db=pubmed&Cmd=Search&Term="Di Rienzo A"%5BAuthor%5D&itool=EntrezSystem2.PEntrez.Pubmed.Pubmed_ResultsPanel.Pubmed_DiscoveryPanel.Pubmed_RVAbstractPlus), [Wilson AC](http://www.ncbi.nlm.nih.gov/sites/entrez?Db=pubmed&Cmd=Search&Term="Wilson AC"%5BAuthor%5D&itool=EntrezSystem2.PEntrez.Pubmed.Pubmed_ResultsPanel.Pubmed_DiscoveryPanel.Pubmed_RVAbstractPlus). 1991. Branching pattern in the evolutionary tree for human mitochondrial DNA. [Proc Natl Acad Sci U S A.](javascript:AL_get(this, 'jour', 'Proc Natl Acad Sci U S A.');)88:1597-601.

Ely B, Wilson JL, Jackson F, Jackson BA. 2006. [African-American mitochondrial DNAs often match mtDNAs found in multiple African ethnic groups.](http://www.ncbi.nlm.nih.gov/pubmed/17038170?ordinalpos=1&itool=EntrezSystem2.PEntrez.Pubmed.Pubmed_ResultsPanel.Pubmed_DefaultReportPanel.Pubmed_RVDocSum) BMC Biol. 4:34.

González AM, Karadsheh N, Maca-Meyer N, Flores C, Cabrera VM, Larruga JM. 2008. [Mitochondrial DNA variation in Jordanians and their genetic relationship to other Middle East populations.](http://www.ncbi.nlm.nih.gov/pubmed/18428014?ordinalpos=15&itool=EntrezSystem2.PEntrez.Pubmed.Pubmed_ResultsPanel.Pubmed_DefaultReportPanel.Pubmed_RVDocSum) Ann Hum Biol. 35:212-31.

Graven L, Passarino G, Semino O, Boursot P, Santachiara-Benerecetti S, Langaney A, Excoffier L. 1995. [Evolutionary correlation between control region sequence and restriction polymorphisms in the mitochondrial genome of a large Senegalese Mandenka sample.](http://www.ncbi.nlm.nih.gov/pubmed/7700157?ordinalpos=4&itool=EntrezSystem2.PEntrez.Pubmed.Pubmed_ResultsPanel.Pubmed_DefaultReportPanel.Pubmed_RVDocSum) Mol Biol Evol. 12:334-45.

Kivisild T, Reidla M, Metspalu E, Rosa A, Brehm A, Pennarun E, Parik J, Geberhiwot T, Usanga E, Villems R. 2004. Ethiopian Mitochondrial DNA Heritage: Tracking Gene Flow Across and Around the Gate of Tears. Am. J. Hum. Genet. 75:752–770.

Knight A, Underhill PA, Mortensen HM, Zhivotovsky LA, Lin AA, Henn BM, Louis D, Ruhlen M, Mountain JL. 2003. [African Y chromosome and mtDNA divergence provides insight into the history of click languages.](http://www.ncbi.nlm.nih.gov/pubmed/12646128?ordinalpos=4&itool=EntrezSystem2.PEntrez.Pubmed.Pubmed_ResultsPanel.Pubmed_DefaultReportPanel.Pubmed_RVDocSum) Curr Biol. 13:464-73.

Krings M, Salem AE, Bauer K, Geisert H, Malek AK, Chaix L, Simon C, Welsby D, Di Rienzo A, Utermann G, Sajantila A, Pääbo S, Stoneking M. 1999. [mtDNA analysis of Nile River Valley populations: A genetic corridor or a barrier to migration?](http://www.ncbi.nlm.nih.gov/pubmed/10090902?ordinalpos=10&itool=EntrezSystem2.PEntrez.Pubmed.Pubmed_ResultsPanel.Pubmed_DefaultReportPanel.Pubmed_RVDocSum) Am J Hum Genet. 64:1166-76.

Mateu E, Comas D, Calafell F, Pérez-Lezaun A, Abade A, Bertranpetit J. 1997. A tale of two islands: population history and mitochondrial DNA sequence variation of Bioko and São Tomé, Gulf of Guinea. Ann Hum Genet. 61:507-18.

Ottoni C, Martínez-Labarga C, Loogväli EL, Pennarun E, Achilli A, De Angelis F, Trucchi E, Contini I, Biondi G, Rickards O. 2009. [First genetic insight into Libyan Tuaregs: a maternal perspective.](http://www.ncbi.nlm.nih.gov/pubmed/19476452?ordinalpos=3&itool=EntrezSystem2.PEntrez.Pubmed.Pubmed_ResultsPanel.Pubmed_DefaultReportPanel.Pubmed_RVDocSum) Ann Hum Genet. 73:438-48.

Pereira L, Macaulay V, Torroni A, Scozzari R, Prata MJ, Amorim A. 2001. [Prehistoric and historic traces in the mtDNA of Mozambique: insights into the Bantu expansions and the slave trade.](http://www.ncbi.nlm.nih.gov/pubmed/11806853?ordinalpos=2&itool=EntrezSystem2.PEntrez.Pubmed.Pubmed_ResultsPanel.Pubmed_DefaultReportPanel.Pubmed_RVDocSum) Ann Hum Genet. 65:439-58.

Plaza S, Calafell F, Helal A, Bouzerna N, Lefranc G, Bertranpetit J, Comas D. 2003. [Joining the pillars of Hercules: mtDNA sequences show multidirectional gene flow in the western Mediterranean.](http://www.ncbi.nlm.nih.gov/pubmed/12914566?ordinalpos=2&itool=EntrezSystem2.PEntrez.Pubmed.Pubmed_ResultsPanel.Pubmed_DefaultReportPanel.Pubmed_RVDocSum) Ann Hum Genet. 67:312-28.

Plaza S, Salas A, Calafell F, Corte-Real F, Bertranpetit J, Carracedo A, Comas D. 2004. Insights into the western Bantu dispersal: mtDNA lineage analysis in Angola. Hum. Genet. 115:439–447.

Quintana-Murci L, Quach H, Harmant C, Luca F, Massonnet B, Patin E, Sica L, Mouguiama-Daouda P, Comas D, Tzur S, Balanovsky O, Kidd KK, Kidd JR, van der Veen L, Hombert JM, Gessain A, Verdu P, Froment A, Bahuchet S, Heyer E, Dausset J, Salas A, Behar DM. 2008. [Maternal traces of deep common ancestry and asymmetric gene flow between Pygmy hunter-gatherers and Bantu-speaking farmers.](http://www.ncbi.nlm.nih.gov/pubmed/18216239?ordinalpos=14&itool=EntrezSystem2.PEntrez.Pubmed.Pubmed_ResultsPanel.Pubmed_DefaultReportPanel.Pubmed_RVDocSum) Proc Natl Acad Sci U S A 105:1596-601.

Rando JC, Pinto F, González AM, Hernández M, Larruga JM, Cabrera VM, Bandelt HJ. 1998. [Mitochondrial DNA analysis of northwest African populations reveals genetic exchanges with European, near-eastern, and sub-Saharan populations.](http://www.ncbi.nlm.nih.gov/pubmed/10363131?ordinalpos=8&itool=EntrezSystem2.PEntrez.Pubmed.Pubmed_ResultsPanel.Pubmed_DefaultReportPanel.Pubmed_RVDocSum) Ann Hum Genet. 62:531-50.

Richards M, Corte-Real H, Forster P, Macaulay V, Wilkinson-Herbots H, Demaine A, Papiha S, Hedges R, Bandelt HJ, Sykes B. 1996. Paleolithic and Neolithic lineages in the European mitochondrial gene pool. Am J Hum Genet 59:185-203.

Richards M, Macaulay V, Hickey E, Vega E, Sykes B, GuidaV, Rengo C, Sellitto D, Cruciani F, Kivisild T, Villems R, Thomas M, Rychkov S, Rychkov O, Rychkov Y, Gölge M, Dimitrov D, Hill E, Bradley D, Romano V, Calì F, Vona G, Demaine A, Papiha S, Triantaphyllidis C, Stefanescu G, Hatina J, Belledi M, Di Rienzo A, Novelletto A, Oppenheim A, Nørby S, Santachiara-Benerecetti S, Scozzari R, Torroni A, Bandelt HJ. 2000. Tracing European founder lineages in the Near Eastern mtDNA pool.  Am J Hum Genet 67, 1251-1276.

Rowold DJ, Luis JR, Terreros MC, Herrera RJ. 2007. [Mitochondrial DNA geneflow indicates preferred usage of the Levant Corridor over the Horn of Africa passageway.](http://www.ncbi.nlm.nih.gov/pubmed/17447003?ordinalpos=1&itool=EntrezSystem2.PEntrez.Pubmed.Pubmed_ResultsPanel.Pubmed_DefaultReportPanel.Pubmed_RVDocSum) J Hum Genet. 52:436-47.

Salas A, Richards M, De la Fe T, Lareu MV, Sobrino B, Sánchez-Diz P, Macaulay V, Carracedo A. 2002. [The making of the African mtDNA landscape.](http://www.ncbi.nlm.nih.gov/pubmed/12395296?ordinalpos=3&itool=EntrezSystem2.PEntrez.Pubmed.Pubmed_ResultsPanel.Pubmed_DefaultReportPanel.Pubmed_RVDocSum) Am J Hum Genet. 71:1082-111.

Watson E, Forster P, Richards M, Bandelt HJ. 1997. Mitochondrial footprints of human expansions in Africa. Am J Hum Genet 61:691–704.
