# Supplementary material for: The trans-Saharan slave trade - clues from interpolation analyses and high-resolution characterization of mitochondrial DNA lineages
Source: BMC Evol Biol. 2010 May 10;10:138. doi: 10.1186/1471-2148-10-138 (PMC2875235; doi:10.1186/1471-2148-10-138)
Supplement: Additional file 3 — Information for samples used in the phylogeny of complete L3 sequences. Information about location, bibliographic reference and GenBank Accession Number for samples used in the phylogeny of complete L3 sequences. [file 1471-2148-10-138-S3.DOC]

Supplementary material 3 - Information for samples used in phylogeny of complete L3 sequences.

| Sample | Location | Reference | Accession Number |
| --- | --- | --- | --- |
| 1 | USA | Herrnstadt et al. (2002) | EF657293 |
| 2 | USA | Herrnstadt et al. (2002) | EF657303 |
| 3 | USA | Herrnstadt et al. (2002) | EF657330 |
| 4 | Morocco | Behar et al. (2008) | EU092814 |
| 5 | Libya | Behar et al. (2008) | EU092682 |
| 6 | Burkina | Kivisild et al. (2006) | DQ112738 |
| 7 | Burkina | Kivisild et al. (2006) | DQ112745 |
| 8 | Guinea Bissau | Behar et al. (2008) | EU092727 |
| 9 | Jordan | Behar et al. (2008) | EU092759 |
| 10 | Egypt | Behar et al. (2008) | EU092767 |
| 11 | Egypt | Behar et al. (2008) | EU092768 |
| 12 | USA | Herrnstadt et al. (2002) | EF657241 |
| 13 | USA | Herrnstadt et al. (2002) | EF657464 |
| 16 | Burkina | Kivisild et al. (2006) | DQ112742 |
| 17 | Burkina | Kivisild et al. (2006) | DQ112743 |
| 18 | Mauritania | Maca-Meyer et al. (2001) | AF381991 |
| 19 | Burkina | Kivisild et al. (2006) | DQ112748 |
| 20 | Guinea Bissau | Behar et al. (2008) | EU092726 |
| 21 | Chad | Behar et al. (2008) | EU092899 |
| 14 | Dominica | Kivisild et al. (2006) | DQ112712 |
| 15 | Ethiopia | Torroni et al. (2006) | DQ341073 |
| 22 | South Africa | Mishmar et al. (2003) | AY195784 |
| 23 | Mozambique | Behar et al. (2008) | EU092694 |
| 24 | Tunisia | Behar et al. (2008) | EU092826 |
| 25 | Jordan | Behar et al. (2008) | EU092920 |
| 26 | USA | Behar et al. (2008) | EU092964 |
| 27 | Libya | Behar et al. (2008) | EU092820 |
| 28 | Chad | Behar et al. (2008) | EU092906 |
| 29 | USA | Herrnstadt et al. (2002) | EF657237 |
| 30 | Dominica | Kivisild et al. (2006) | DQ112711 |
| 31 | Pakistan | Behar et al. (2008) | EU092932 |
| 32 | Kuwait | Behar et al. (2008) | EU092779 |
| 33 | Pakistan | Behar et al. (2008) | EU092933 |
| 34 | Morocco | Behar et al. (2008) | EU092815 |
| 35 | Gambia | Behar et al. (2008) | EU092960 |
| 36 | Yemen | Behar et al. (2008) | EU092795 |
| 37 | Tunisia | Behar et al. (2008) | EU092825 |
| 38 | Ethiopia | Behar et al. (2008) | EU092669 |
| 39 | Syria | Behar et al. (2008) | EU092744 |
| 40 | Dominica | Kivisild et al. (2006) | DQ112746 |
| 41 | Burkina | Kivisild et al. (2006) | DQ112710 |
| 42 | Guinea Bissau | Behar et al. (2008) | EU092725 |
| 43 | Yemen | Behar et al. (2008) | EU092660 |
| 44 | Ethiopia | Torroni et al. (2006) | DQ341074 |
| 45 | West Africa | Kivisild et al. (2006) | DQ112884 |
| 46 | Yemen | Behar et al. (2008) | EU092796 |
| 47 | Mozambique | Behar et al. (2008) | EU092702 |
| 48 | West Africa | Ingman et al. (2000) | AF347014 |
| 49 | Yemen | Behar et al. (2008) | EU092797 |
| 50 | Kenya | Behar et al. (2008) | EU092910 |
| 51 | Pakistan | Behar et al. (2008) | EU092934 |
| 52 | Burkina | Kivisild et al. (2006) | DQ112744 |
| 53 | Chad | Behar et al. (2008) | EU092878 |
| 54 | Burkina | Kivisild et al. (2006) | DQ112739 |
| 55 | Chad | Behar et al. (2008) | EU092901 |
| 56 | Dominica | Kivisild et al. (2006) | DQ112715 |
| 57 | Ethiopia | Torroni et al. (2006) | DQ341072 |
| 58 | USA | Herrnstadt et al. (2002) | EF657544 |
| 59 | Guinea Bissau | Behar et al. (2008) | EU092728 |
| 60 | Egypt | Behar et al. (2008) | EU092762 |
| 61 | USA | Herrnstadt et al. (2002) | EF657277 |
| 62 | Tunisia | Behar et al. (2008) | EU092830 |
| 63 | South Africa | Mishmar et al. (2003) | AY195782 |
| 64 | Dominica | Kivisild et al. (2006) | DQ112714 |
| 74 | Guinea Bissau | Behar et al. (2008) | EU092735 |
| 65 | Jordan | Maca-Meyer et al. (2001) | AF381998 |
| 66 | Dominica | Kivisild et al. (2006) | DQ112716 |
| 67 | South Africa | Kivisild et al. (2006) | DQ112857 |
| 68 | South Africa | Behar et al. (2008) | EU092853 |
| 69 | South Africa | Kivisild et al. (2006) | DQ112847 |
| 70 | South Africa | Kivisild et al. (2006) | DQ112851 |
| 71 | South Africa | Kivisild et al. (2006) | DQ112853 |
| 72 | Dominica | Kivisild et al. (2006) | DQ112713 |
| 73 | Burkina | Kivisild et al. (2006) | DQ112756 |
| 75 | USA | Herrnstadt et al. (2002) | EF657580 |
| 76 | Syria | Behar et al. (2008) | EU092742 |
| 77 | Kenya | Kivisild et al. (2006) | DQ112949 |
| 78 | Chad | Behar et al. (2008) | EU092900 |
| 79 | Israel | Behar et al. (2008) | EU092685 |
| 80 | Jordan | Behar et al. (2008) | EU092757 |
| 81 | USA | Herrnstadt et al. (2002) | EF657361 |
| 82 | Tunisia | Behar et al. (2008) | EU092827 |
| 83 | Chad | Behar et al. (2008) | EU092889 |
| 84 | USA | Herrnstadt et al. (2002) | EF657356 |
| 85 | USA | Herrnstadt et al. (2002) | EF657550 |
| 86 | USA | Herrnstadt et al. (2002) | EF657362 |
| 87 | USA | Herrnstadt et al. (2002) | EF657297 |
| 88 | South Arabia | Behar et al. (2008) | EU092749 |
| 89 | South Africa | Behar et al. (2008) | EU092849 |
| 90 | South Africa | Behar et al. (2008) | EU092877 |
| 91 | Dominica | Kivisild et al. (2006) | DQ112719 |
| 92 | South Africa | Behar et al. (2008) | EU092869 |
| 93 | USA | Behar et al. (2008) | EU092962 |
| 94 | Mozambique | Behar et al. (2008) | EU092709 |
| 95 | Oman | Behar et al. (2008) | EU092790 |
| 96 | Syria | Behar et al. (2008) | EU092740 |
| 97 | Chad | Behar et al. (2008) | EU092881 |
| 98 | Chad | Behar et al. (2008) | EU092882 |
| 99 | Chad | Behar et al. (2008) | EU092887 |
| 100 | Mozambique | Behar et al. (2008) | EU092693 |
| 101 | South Africa | Behar et al. (2008) | EU092864 |
| 102 | Kenya | Behar et al. (2008) | EU092917 |
| 103 | West Africa | Ingman et al. (2000) | AF346980 |
| 104 | Mozambique | Behar et al. (2008) | EU092698 |
| 105 | Israel | Behar et al. (2008) | EU092675 |
| 106 | Israel | Behar et al. (2008) | EU092681 |
| 107 | Egypt | Behar et al. (2008) | EU092769 |
| 108 | Tunisia | Behar et al. (2008) | EU092928 |
| 109 | Burkina | Kivisild et al. (2006) | DQ112726 |
| 110 | Burkina | Kivisild et al. (2006) | DQ112728 |
| 111 | Burkina | Kivisild et al. (2006) | DQ112729 |
| 112 | USA | Herrnstadt et al. (2002) | EF657338 |
| 113 | Guinea Bissau | Behar et al. (2008) | EU092729 |
| 114 | USA | Herrnstadt et al. (2002) | EF657255 |
| 115 | Burkina | Kivisild et al. (2006) | DQ112740 |
| 116 | Dominica | Kivisild et al. (2006) | DQ112721 |
| 117 | Ethiopia | Torroni et al. (2006) | DQ341071 |
| 118 | Zaire | Ingman et al. (2000) | AF346994 |
| 119 | USA | Herrnstadt et al. (2002) | EF657272 |
| 120 | USA | Herrnstadt et al. (2002) | EF657305 |
| 121 | USA | Herrnstadt et al. (2002) | EF657256 |
| 122 | Guinea Bissau | Behar et al. (2008) | EU092730 |
| 123 | Jordan | Behar et al. (2008) | EU092919 |
| 124 | West Africa | Ingman et al. (2000) | AF347015 |
| 125 | Burkina | Kivisild et al. (2006) | DQ112734 |
| 126 | Oman | Behar et al. (2008) | EU092784 |
| 127 | Oman | Behar et al. (2008) | EU092785 |
| 128 | Dominica | Kivisild et al. (2006) | DQ112720 |
| 129 | Egypt | Behar et al. (2008) | EU092777 |
| 130 | USA | Herrnstadt et al. (2002) | EF657321 |
| 131 | Mozambique | Behar et al. (2008) | EU092706 |
| 132 | Oman | Behar et al. (2008) | EU092789 |
| 133 | Kenya | Behar et al. (2008) | EU092909 |
| 134 | Israel | Behar et al. (2008) | EU092677 |
| 135 | West Africa | Ingman et al. (2000) | AF346967 |
| 136 | USA | Herrnstadt et al. (2002) | EF657390 |
| 137 | Burkina | Kivisild et al. (2006) | DQ112747 |
| 138 | Chad | Behar et al. (2008) | EU092897 |
| 139 | Lebanon | Behar et al. (2008) | EU092752 |
| 140 | Dominica | Kivisild et al. (2006) | DQ112722 |
| 141 | Mozambique | Behar et al. (2008) | EU092695 |
| 142 | Guinea Bissau | Behar et al. (2008) | EU092731 |
| 143 | USA | Herrnstadt et al. (2002) | EF657302 |
| 144 | Burkina | Kivisild et al. (2006) | DQ112757 |
| 145 | Egypt | Behar et al. (2008) | EU092776 |
| 146 | Libya | Behar et al. (2008) | EU092821 |
| 147 | Ethiopia | Torroni et al. (2006) | DQ341070 |
| 148 | USA | Behar et al. (2008) | EU092961 |
| 149 | Sudan | Kivisild et al. (2006) | DQ112958 |
| 150 | Sudan | Kivisild et al. (2006) | DQ112960 |
| 151 | Yemen | Behar et al. (2008) | EU092925 |
| 152 | Ethiopia | Torroni et al. (2006) | DQ341069 |
| 153 | Oman | Behar et al. (2008) | EU092788 |
| 154 | Ethiopia | Torroni et al. (2006) | DQ341068 |
| 155 | Algeria | Behar et al. (2008) | EU092818 |
| 156 | Ethiopia | Behar et al. (2008) | EU092946 |
| 157 | Ethiopia | Torroni et al. (2006) | DQ341066 |
| 158 | Israel | Behar et al. (2008) | EU092684 |
| 159 | Kuwait | Behar et al. (2008) | EU092922 |
| 160 | Yemen | Behar et al. (2008a) | EF556171 |
| 161 | Ethiopia | Behar et al. (2008) | EU092666 |
| 162 | Ethiopia | Torroni et al. (2006) | DQ341067 |
| 163 | Libya | Behar et al. (2008) | EU092822 |
| 164 | Tunisia | Behar et al. (2008) | EU092824 |
| 165 | Chad | Behar et al. (2008) | EU092893 |
| 166 | Ethiopia | Torroni et al. (2006) | DQ341078 |
| 167 | USA | Herrnstadt et al. (2002) | EF657250 |
| 168 | USA | Herrnstadt et al. (2002) | EF657252 |
| 169 | Mozambique | Behar et al. (2008) | EU092696 |
| 170 | South Africa | Behar et al. (2008) | EU092859 |
| 171 | Egypt | Behar et al. (2008) | EU092771 |
| 172 | USA | Herrnstadt et al. (2002) | EF657251 |
| 173 | Burkina | Kivisild et al. (2006) | DQ112736 |
| 174 | Guinea Bissau | Behar et al. (2008) | EU092732 |
| 175 | Israel | Behar et al. (2008) | EU092680 |
| 176 | Lebanon | Behar et al. (2008) | EU092751 |
| 177 | Yemen | Behar et al. (2008) | EU092805 |
| 178 | Jordan | Behar et al. (2008) | EU092758 |
| 179 | USA | Behar et al. (2008) | EU092955 |
| 180 | Ethiopia | Torroni et al. (2006) | DQ341077 |
| 181 | Syria | Behar et al. (2008) | EU092741 |
| 182 | USA | Herrnstadt et al. (2002) | EF657257 |
| 183 | USA | Herrnstadt et al. (2002) | EF657253 |
| 184 | Chad | Behar et al. (2008) | EU092885 |
| 185 | Mozambique | Behar et al. (2008) | EU092704 |
| 186 | South Africa | Behar et al. (2008) | EU092867 |
| 187 | Dominica | Kivisild et al. (2006) | DQ112717 |
| 188 | Oman | Behar et al. (2008) | EU092791 |
| 189 | Kenya | Behar et al. (2008) | EU092914 |
| 190 | Ethiopia | Torroni et al. (2006) | DQ341076 |
| 191 | Sudan | Kivisild et al. (2006) | DQ112956 |
| 192 | Egypt | Behar et al. (2008) | EU092770 |
| 193 | Chad | Behar et al. (2008) | EU092879 |
| 194 | Chad | Behar et al. (2008) | EU092896 |
| 195 | Ethiopia | Torroni et al. (2006) | DQ341075 |
| 196 | Ethiopia | Torroni et al. (2006) | DQ341080 |
| 197 | Sudan | Kivisild et al. (2006) | DQ112957 |
| 198 | Sudan | Kivisild et al. (2006) | DQ112961 |
| 199 | Lebanon | Behar et al. (2008) | EU092753 |
| 200 | Egypt | Behar et al. (2008) | EU092772 |
| 201 | East Africa | Ingman et al. (2000) | AF347000 |
| 202 | Ethiopia | Behar et al. (2008a) | EF556166 |
| 203 | Yemen | Behar et al. (2008) | EU092798 |
| 204 | Guinea Bissau | Behar et al. (2008) | EU092736 |
| 205 | Tunisia | Behar et al. (2008) | EU092828 |
| 206 | Ethiopia | Behar et al. (2008) | EU092949 |
| 207 | Chad | Behar et al. (2008) | EU092888 |
| 208 | Chad | Behar et al. (2008) | EU092905 |
| 209 | Ethiopia | Torroni et al. (2006) | DQ341079 |
| 210 | Dominica | Kivisild et al. (2006) | DQ112718 |
| 211 | Ethiopia | Torroni et al. (2006) | DQ341081 |
| 212 | Ethiopia | Behar et al. (2008) | EU092943 |
| 213 | Chad | Cerný et al. (2009) | FJ625845 |
| 214 | Chad | Cerný et al. (2009) | FJ625860 |
| 215 | Chad | Cerný et al. (2009) | FJ625856 |
| 216 | Chad | Cerný et al. (2009) | FJ625858 |
| 217 | Chad | Cerný et al. (2009) | FJ625850 |
| 218 | Chad | Cerný et al. (2009) | FJ625859 |
| 219 | Chad | Cerný et al. (2009) | FJ625852 |
| 220 | Chad | Cerný et al. (2009) | FJ625857 |
| 221 | Chad | Cerný et al. (2009) | FJ625851 |
| 222 | Chad | Cerný et al. (2009) | FJ625855 |
| 223 | Chad | Cerný et al. (2009) | FJ625854 |
| 224 | Chad | Cerný et al. (2009) | FJ625846 |
| 225 | Chad | Cerný et al. (2009) | FJ625847 |
| 226 | Chad | Cerný et al. (2009) | FJ625853 |
| 227 | Chad | Cerný et al. (2009) | FJ625849 |
| 228 | Chad | Cerný et al. (2009) | FJ625848 |
| 229 | Tunisia | Costa et al. (2009) | FJ460529 |
| 230 | Tunisia | Costa et al. (2009) | FJ460536 |
| 231 | Tunisia | Costa et al. (2009) | FJ460540 |
| 232 | Tunisia | Costa et al. (2009) | FJ460533 |
| 233 | Egypt | Kujanová et al. (2009) | EU935449 |
| 234 | Egypt | Kujanová et al. (2009) | EU935440 |
| 235 | Egypt | Kujanová et al. (2009) | EU935465 |
| 236 | Egypt | Kujanová et al. (2009) | EU935451 |
| J70 | Morocco | present study | XXXXXXX |
| J71 | Morocco | present study | XXXXXXX |
| J73 | Morocco | present study | XXXXXXX |
| J74 | Morocco | present study | XXXXXXX |
| J77 | Morocco | present study | XXXXXXX |
| J78 | Morocco | present study | XXXXXXX |
| J80 | Morocco | present study | XXXXXXX |
| J81 | Morocco | present study | XXXXXXX |

Behar DM, Villems R, Soodyall H, Blue-Smith J, Pereira L, Metspalu E, Scozzari R, Makkan H, Tzur S, Comas D, Bertranpetit J, Quintana-Murci L, Tyler-Smith C, Wells RS, Rosset S, Genographic Consortium. 2008. The dawn of human matrilineal diversity. Am J Hum Genet 82:1130-1140.

Behar DM, Metspalu E, Kivisild T, Rosset S, Tzur S, Hadid Y, Yudkovsky G, Rosengarten D, Pereira L, Amorim A, Kutuev I, Gurwitz D, Bonne-Tamir B, Villems R, Skorecki K.2008a. Counting the founders: the matrilineal genetic ancestry of the Jewish Diaspora. PLoS ONE 3:E2062.

Cerný V, Fernandes V, Costa MD, Hajek M, Mulligan CJ, Pereira L. 2009. Migration of Chadic speaking pastoralists within Africa based on population structure of Chad Basin and phylogeography of mitochondrial L3f haplogroup. BMC Evol. Biol. 9:63.

Costa MD, Cherni L, Fernandes V, Freitas F, el Gaaied ABA, Pereira L. 2009. Data from complete mtDNA sequencing of Tunisian centenarians:testing haplogroup association and the 'golden mean' to longevity. Mech Ageing Dev 130:222-226.

Herrnstadt C, Elson JL, Fahy E, Preston G, Turnbull DM, Anderson C, Ghosh SS, Olefsky JM, Beal MF, Davis RE, Howell N. 2002. Reduced-median-network analysis of complete mitochondrial DNA coding-region sequences for the major African, Asian, and European haplogroups. Am J Hum Genet 70:1152-1171.

Ingman M, Kaessmann H, Paabo S, Gyllensten U. 2000. Mitochondrial genome variation and the origin of modern humans. Nature 408:708-713.

Kivisild T, Shen P, Wall DP, Do B, Sung R, Davis K, Passarino G, Underhill PA, Scharfe C, Torroni A, Scozzari R, Modiano D, Coppa A, de Knijff P, Feldman M, Cavalli-Sforza LL, Oefner PJ. 2006. The role of selection in the evolution of human mitochondrial genomes. Genetics 172:373-387.

Kujanová M, Pereira L, Fernandes V, Pereira JB, Cerny V. 2009. Predominantly neolithic contribution to the population history of the Egyptian Western Desert; mtDNA and Y chromosome analyses from the oasis of el-Hayez. Am J Phy Anthropol 140:336-346.

Maca-Meyer N, Gonzalez AM, Larruga JM, Flores, Cabrera VM. 2001. Major genomic mitochondrial lineages delineate early human expansions. BMC Genet. 2:13.

Mishmar D, Ruiz-Pesini E, Golik P, Macaulay V, Clark AG, Hosseini S, Brandon M, Easley K, Chen E, Brown MD, Sukernik RI, Olckers A, Wallace DC. 2003. Natural selection shaped regional mtDNA variation in humans. Proc. Natl. Acad. Sci. U.S.A. 100:171-176.

Torroni A, Achilli A, Macaulay V, Richards M, Bandelt HJ. 2006. Harvesting the fruit of the human mtDNA tree. Trends Genet. 22:339-345.
